# Vertical and Horizontal Transmission of Neosporosis in Three Consecutive Pregnancies of Naturally Infected Pregnant Cows and the Effect of Vaccination on Abortion Rates

**DOI:** 10.3390/vaccines13020131

**Published:** 2025-01-27

**Authors:** Sharon Tirosh-Levy, Elena Blinder, Daniel Yasur-Landau, Yaniv Lavon, Jacob Joost Doekes, Monica L. Mazuz

**Affiliations:** 1 Division of Parasitology, Kimron Veterinary Institute, Beit Dagan 502001, Israel; sharontirosh@gmail.com (S.T.-L.); elenab@moag.gov.il (E.B.); daniely@moag.gov.il (D.Y.-L.); 2 Koret School of Veterinary Medicine, The Robert H. Smith Faculty of Agriculture, Food and Environment, The Hebrew University of Jerusalem, Rehovot 7610001, Israel; 3 Israel Cattle Breeders Association, Caesarea 3097020, Israel; yaniv@icba.co.il; 4 Hachaklait Veterinary Services, Caesarea 3097020, Israel; dukas@hak.org.il

**Keywords:** *Neospora caninum*, neosporosis, abortion, cattle, vaccination, control, vertical transmission, horizontal transmission

## Abstract

Background/Objectives:Neosporosis is a major cause of abortions in cattle worldwide. Primary results showed that the administration of a live attenuated vaccine during the mid-pregnancy stage of naturally infected cows may assist in preventing abortions. In this study, the effect of vaccination was evaluated in five dairy herds, with a follow-up of three consecutive pregnancies and re-vaccination during the subsequent pregnancies of some of the cows. Methods: A total of 1059 heifers were serologically tested during their first pregnancy, and 260 and 21 of them were re-tested during their second and third pregnancies. Vaccination was administered to 193 of 420 cows with antibody titers of 1:800 or higher, and 23 of them were re-vaccinated. Data were collected regarding the outcome of each pregnancy, the number of inseminations required and removal from the herd. Vertical transmission was evaluated in 136 pre-colostral calves born from 29 vaccinated and 107 unvaccinated dams. Results: The total seroprevalence using a cutoff titer of 1:800 was 33.1, 36.5 and 85.7% during the three consecutive pregnancies. The antibody titers of individual cows fluctuated over time. Abortion rates and the rate of removal from the herd were significantly higher in seropositive cows. The rate of vertical transmission increased in correlation with the dam’s antibody titer. Immunization resulted in lower abortion rates at two of the farms. Vaccine efficacy ranged from a negative effect to 54% at different farms, with an overall efficacy of 10.4%. The effect of vaccination on abortions, reproductive performance, antibody titers, vertical transmission and removal from the herd was not significant. Conclusions: These results demonstrate varying vaccine efficacies among farms and suggest that neosporosis is a multifactorial disease that cannot be solely controlled by vaccination.

## 1. Introduction

*Neospora caninum* is a cyst-forming coccidia, an intracellular parasite with a heteroxenous life cycle that includes canids as its definitive host and a wide variety of mammalian species as its intermediate hosts [1,2]. Most intermediate hosts do not exhibit clinical signs, and the main clinical consequence of *Neospora* infection is an increased risk of abortion [1]. Neosporosis has been reported in most parts of the word with varying prevalence, and its main economic significance is in its role as a cause of abortions in cattle [3].

Cattle may become infected with *Neospora* via horizonal transmission, that is, through the ingestion of oocysts secreted by definitive canid hosts [1,4]. However, vertical transmission is considered the main route of transmission in cattle. Thus, high rates of pregnant infected cows that do not experience abortion lead to the calving of apparently healthy infected calves [4,5,6,7].

These calves become *Neospora* carrier cows, which have an increased risk of abortions [8] and repeated abortions in subsequential pregnancies [5]. The risk of abortion increases in correlation with the anti-*Neospora* antibody titer [5,9,10]. It has been suggested that immunosuppression during pregnancy may lead to the re-emergence of parasites from tissues cysts and transient parasitemia, which may infect the fetus [11]. Its high rates of vertical transmission in combination with its long-term effect on reproductive performance may lead to an increased prevalence and cumulative economic effect of neosporosis in some herds, making prevention and control attempts challenging.

No effective chemotherapy or commercial vaccine is currently available for neosporosis. Since *Neospora* is an intracellular parasite, effective immune response is based mainly on cell-mediated immunity, while the presence of specific antibodies has not proven to be protective [12,13]. Several attempts have been made to produce an effective vaccine against neosporosis using specific antigens, tachyzoite lysate or live tachyzoites, with part of them showing promising preliminary results in mice and cattle [14,15,16,17,18,19,20,21]. However, a previous commercial vaccine, Bovilis^®^ Neoguard, a killed *Neospora caninum* tachyzoite vaccine (Intervet International B.V., Boxmeer, The Netherlands), only demonstrated moderate protection against abortions and was discontinued [22,23].

Recently, a live attenuated vaccine was developed using the Israeli *N. caninum* isolate (NcIs491) [24]. This vaccine has been evaluated using a vaccination approach where only seropositive cows were vaccinated at mid-pregnancy (between 120 and 140 days of pregnancy). This approach aimed to increase immune protection at the stage of immunomodulation during pregnancy in order to prevent parasite re-emergence and any risk of infection of the fetus. The preliminary results under field conditions demonstrated an efficacy of 39% in preventing abortions when using fresh–live vaccination [25], and an efficacy of 28.4% when using a frozen–live vaccine [9]. In the latter study, the live vaccine was kept frozen in liquid nitrogen to provide easier storage and use in the field, which could be implemented in the future by veterinary practitioners. The efficacy of the frozen–live vaccine varied between farms, suggesting that environmental and husbandry factors may affect the efficacy of vaccination in preventing abortions.

The current study was set to re-evaluate the efficacy of frozen–live vaccination during pregnancy in preventing abortions, as well as to evaluate whether single vaccination has a long-term effect on more than one pregnancy or if re-vaccination is necessary during each pregnancy. Therefore, this study was designed as a long-term study following the same cohort of cows during three consecutive pregnancies, evaluating *Neospora* serological status at each pregnancy, vaccinating seropositive cows and documenting the outcome of each pregnancy.

## 2. Materials and Methods

### 2.1. Study Design

The farms that participated in this study all had a history of neosporosis, which was confirmed and evaluated by a serological diagnosis of a sample of the cows in each herd. Farms were selected according to the cooperation of the farmers and the attending veterinarians. All farms used computerized monitoring for their animals, which made all the relevant data available. This study was conducted at five farms between November 2020 and May 2023. One of the farms (Farm 5) had also been included in the preliminary study, with a vaccine efficacy (VE) of 75% [9]. One farm (Farm 4) was removed from the study due to a lack of cooperation. Another farm (Farm 6) was added to the study in May 2022 in place of Farm 4.

The study population comprised 1059 heifers, between 116 and 450 from each farm, which were first sampled on day 110–120 of their first pregnancy and re-sampled in the same timeframe during their consecutive pregnancy. The timing of sample collection was selected to represent the stage of the pregnancy in which physiological immunosuppression may evoke the re-emergence of parasites and an increase in antibody titer, which reflects the risk of abortion. In addition, sampling took place following the confirmation of pregnancy at the end of the first trimester [11]. Cows were screened for serologic exposure to *Neospora* spp. Using the indirect fluorescent antibody test (IFAT). Since parasitemia is short and transient, the antibody levels reflect exposure to *Neospora*, and generally correspond to parasite load. Only seropositive cows with antibody titers higher than 1:800 were considered for vaccination, as this is the antibody titer which was previously demonstrated to be associated with an increased chance of abortion [5].

**Figure 1:**
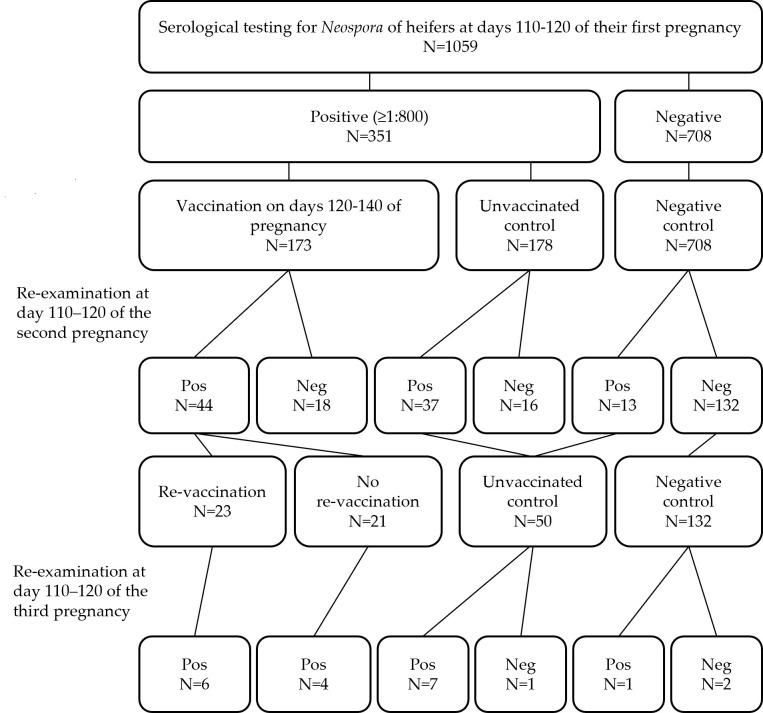
The study design, including the number of cows included in each group.

The seropositive heifers at each farm were divided into two groups. The first group was vaccinated with *N. caninum* live frozen tachyzoites on days 120–140 of pregnancy, while the second group served as unvaccinated controls and no treatment was administered. All cows in the unvaccinated group remained unvaccinated throughout the study period. Half of the vaccinated cows were re-vaccinated during their second pregnancy, while the other half were not re-vaccinated (Figure 1).

Data were collected regarding the outcome of all pregnancies, the number of inseminations required to achieve each pregnancy and the date and reason for removal from the herd (when relevant). To evaluate the rate of vertical transmission, two of the farms (Farms 1 and 2) collected blood samples from the calves born to the cows included in this study prior to their ingestion of colostrum to further the serological analyses, as the bovine placenta is impermissible to the transfer of antibodies [26].

This study was conducted upon the owners’ consent and was approved by the Animal Experiments Welfare Committee of the Kimron Veterinary Institute (020_b18181_6).

### 2.2. Sample Collection and Serological Testing

Pregnancy examinations were performed on days 110 to 120 after insemination by the attending veterinarians using the fetal membrane slip method, and blood was collected from the tail vein of all pregnant cows into a sterile serum collection tube without anticoagulants. Blood was collected from the jugular vein of newborn calves prior to colostrum feeding. All blood samples were centrifuged at 4000× *g* for 4 min and the serum was separated. The presence of anti-*Neospora* spp. antibodies was determined by an immunofluorescence antibody test (IFAT), as previously described [27]. The antigen was prepared in-house using culture-derived NC-1 tachyzoites with an anti-cattle IgG secondary antibody (A8917, Sigma-Aldrich, Darmstadt, Germany), and the results were interpreted using a fluorescent microscope. The highest dilution of serum exhibiting fluorescence of the whole *Neospora* organism was considered as the endpoint titer. An antibody titer of 1:200 was considered as borderline, and antibody titers of 1:800 or higher were considered positive, in concurrence with previous studies [5,9,25].

### 2.3. Vaccination Procedure

Vaccination was performed with doses of 2 × 10^8^ live *Neospora caninum* tachyzoites administered subcutaneously on days 120–140 post-insemination. Parasite culture and vaccine preparation were performed as previously described [9,25]. Vaccine doses were kept frozen in liquid nitrogen until use.

### 2.4. Statistical Analysis

The agreement between the serological status of two consecutive pregnancies was evaluated using Cohen’s kappa.

*Neospora* seroprevalence was compared between pregnancies. For the population of unvaccinated cows, associations between *Neospora* serological status and the rate of abortions, the rate of multiple inseminations, the rate of cows removed from the herd and the rate of cows removed from the herd due to reproduction failure to the total cows removed during each pregnancy were evaluated. The effect of the vaccination of cows with antibody titers of 1:800 or higher on abortion, the rate of multiple inseminations, the rate of cows removed from the herd and the rate of cows removed from the herd due to reproduction failure to the total cows removed during each pregnancy were evaluated. These analyses were performed using the generalized estimating equation (GEE) with a logit link function, with each sample defined as the subject and the cow as within subject effect.

The association between vaccination and vertical transmission and the effect of repeated vaccination on abortion rates was evaluated using Fisher’s exact test and the odds ratio (OR). The correlation between the number of pregnancies and the abortion rate was estimated by Spearman’s rho (ρ).

The effect of vaccination on the abortion rates in different farms was evaluated in the population of cows with anti-*Neospora* antibody titers of 1:800 or higher using Fisher’s exact test and the odds ratio (OR). Vaccine efficacy (VE) was calculated using the following formula: VE = (ARU–ARV)/ARU × 100 (ARU = abortion rate in unvaccinated cows; ARV = abortion rate in vaccinated cows).

The statistical significance of all analyses was set at *p* < 0.05. The analysis was performed using SPSS 27.0^®^ (IBM Corp, Armonk, NY, USA) and Win Pepi 11.43^®^ statistical software (Abramson, J.H. WINPEPI updated: computer programs for epidemiologists and their teaching potential. Epidemiologic Perspectives & Innovations, 2011, 8:1).

## 3. Results

### 3.1. Neospora Seroprevalence in Heifers

**Figure 2:**
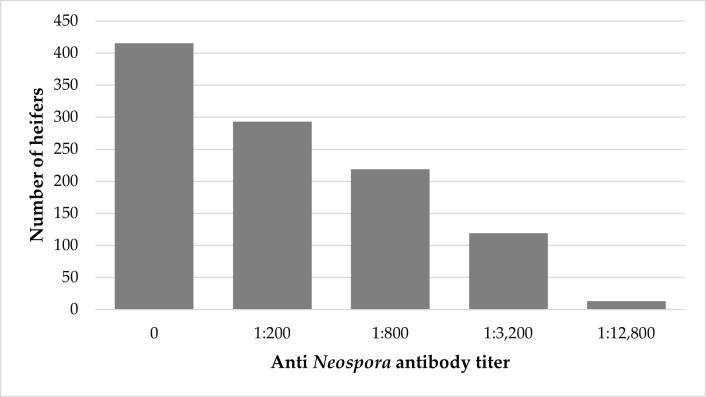
The distribution of antibody titers in 1059 heifers sampled on days 110–120 of their first pregnancy.

A total of 1059 heifers were sampled during their first pregnancy (Table 1), with 116 to 450 tested at each farm. The antibody titers on days 110–120 of the first pregnancy ranged between 0 and 1:12,800, with a median of 200 and an interquartile range (IQR) of 800. The overall seroprevalence of neosporosis in heifers, including heifers with borderline titers, was 60.8% [644 of 1059; 95% confidence interval (CI): 57.8–63.77], and ranged between 48 and 83% in different farms. Among them, 293 had a borderline titer of 1:200 (27.7%), while 33.1% had titers of 800 or higher (95% CI: 30.3–36.1, Figure 2), between 18 and 63% in each farm. Out of the 351 seropositive heifers, 173 were vaccinated on days 120–140 of the same pregnancy (Table 1).

### 3.2. Dynamics of Anti-Neospora Serological Titers in Repeated Samples of Individual Cows

Out of the 1059 heifers that were sampled during their first pregnancy, 260 were sampled at least twice and 21 of these were sampled three times. Out of these 260 cows, 116 had a titer of 1:800 or higher during their first pregnancy, 62 of which were vaccinated. A total of 198 unvaccinated cows were re-sampled during one consecutive pregnancy and 11 were sampled during two additional pregnancies.

Among the seronegative heifers (n = 83), 63.8% remained negative in their second pregnancy, while 31.3% seroconverted to a borderline titer and 4.8% became seropositive. Of the heifers with a borderline titer, 26.2% maintained the same antibody titer, while 59.0% tested negative and 14.7% showed an increase in their antibody titer to at least 1:800. Lastly, of the heifers that tested with a high antibody titer of at least 1:800, 68.5% maintained their high antibody titer, 16.7% had a decreased antibody titer of 1:200 (borderline titer) and 14.8% tested negative during their second pregnancy (Table 2).

When considering the borderline titer of 1:200 as a cutoff, the agreement between the serological results on the two consecutive pregnancies was fair (Cohen’s kappa = 0.32; 68.46% agreement). When setting the cutoff at a titer of 1:800, the agreement between the serological results on the two consecutive pregnancies was substantial (Cohen’s kappa = 0.63; 81.92% agreement).

Exploring the fluctuations in antibody titers in the 21 cows that were sampled during three pregnancies revealed that most cows maintained their serological status (negative, borderline or positive); however, the titers did fluctuate between pregnancies, including cases of seroconversion from negative to positive or from positive to negative (Table 3).

Out of the 62 vaccinated cows that were sampled twice, 10 (16.1%) had an increase in their antibody titer in their next pregnancy, the titer of 24 cows (38.7%) remained constant, and 28 cows (45.2%) had a decrease in their antibody titer. Out of these, nine cows (14.5%) dropped to a borderline titer and another nine (14.5%) were seronegative in their next pregnancy following vaccination. The distribution of antibody titers during the second pregnancy did not differ between seropositive cows vaccinated during their previous pregnancy and unvaccinated seropositive cows (*p* = 0.662).

### 3.3. Vertical Transmission

Blood samples were collected from 136 calves of the cows participating in this study before ingestion of colostrum (Table 4). There was a significant positive correlation between the antibody titers of dams and their calves (ρ = 0.679, *p* < 0.001). The incidence of seropositive calves born to a seronegative dam was very low (3.2%, 1 of 31 of seronegative dams), and it had a borderline titer. The rate of vertical transmission among dams with a borderline antibody titer (1:200) was 37.2% (16 of 43 calves, of which 6 presented a borderline titer and 10 had high antibody titers). The rate of vertical transmission from dams with high antibody titers (≥1:800) was 87.1% (54 of 62 calves), with three presenting a borderline titer and the majority presenting an antibody titer 1:800 or higher (51 calves, 82.3%). Of the 62 dams with high antibody titers, 29 were vaccinated during their current pregnancy and 33 were not vaccinated. Of the 33 calves of the unvaccinated dams, 6 were seronegative (18.2%), 1 had a titer of 1:200 (3.0%) and the rest had a titer of at least 1:800 (26 calves, 78.8%). Among the calves of the vaccinated dams, two were seronegative (6.9%), two had an antibody titer of 1:200 (6.9%) and the rest had a titer of at least 1:800 (25 calves, 86.2%). There was no significant difference in vertical transmission rates between the vaccinated and unvaccinated dams when considering a diagnostic cutoff of 1:200 (*p* = 0.264) or when setting it at 1:800 (*p* = 0.445). In addition, there was no significant difference in the distribution of the calf antibody titers from vaccinated and unvaccinated dams (*p* = 0.698).

Five of the dams had twins. Of them, three negative dams had only negative calves. A dam with a titer of 1:800 (not vaccinated) had two calves with a titer of 1:12,800. A dam with a titer of 1:3200 (not vaccinated) had one calf with a titer of 1:800 and another with a titer of 1:3200. Each of these cases was only included in the Table 4 as one incidence, using the higher calf titer.

### 3.4. Reproduction Parameters and Removal from the Herd

Data were obtained regarding the 2027 pregnancies of the 1059 cows included in this study. Of these, blood samples were collected in 1342 pregnancies and the outcome of the pregnancy (normal calving or abortion) was recorded for 1475 of the pregnancies. The data presented here did not differentiate between seropositive vaccinated and unvaccinated cows as the vaccine was not associated with any of these parameters (Section 3.6).

The reported number of inseminations required to achieve each pregnancy ranged between one and eleven (median = 2; IQR = 3). Pregnancies with up to two inseminations were considered as “normal”, and three inseminations or more were considered as “multiple inseminations”, which may suggest difficulty to conceive. Of 2024 pregnancies, 1161 (57.3%) had less than three inseminations, while 862 (42.6%) had multiple inseminations. The rate of multiple insemination in the cows’ first four pregnancies was significantly higher in the second pregnancy (50.4%) than the first (36.6%); however, the third and fourth pregnancies did not differ significantly from the first two (Table 5).

A total of 1302 of the 1475 pregnancies with recorded outcome resulted in normal calving (88.3%), while 173 (11.7%) resulted in abortion. In 53 cases, a history of previous abortion was recorded prior to first sampling (therefore, these cows were still considered as heifers). The rate of abortions increased from 8.5% during the first pregnancy to 46.2% in the third pregnancy and was significantly higher during each consecutive pregnancy (all *p* < 0.001, Table 6). A positive correlation was found between the number of pregnancies and the rate of abortions (ρ = 0.171, *p* < 0.001).

Two hundred and four cows (19.3%) were removed from the herd during the study period. The reasons for removal from the herd were diverse. A total of 6 cows were reported as “dead”, 14 were sold for economic purposes, 10 suffered from infectious diseases (paratuberculosis, mycoplasmosis, bovine ephemeral fever), 6 suffered from trauma, 41 had low milk production and others had various orthopedic, metabolic or other veterinary conditions. A total of 60 of the cows (29.4%) were removed for reasons relating to reproductive problems, including 35 due to abortion, 2 following dystocia and 23 on account of failure to conceive. Of the total cows removed from the herd, 100 (49.0%) were removed during their first pregnancy, 97 (47.5%) during their second pregnancy, and 7 (3.4%) during their third pregnancy. Of the 60 cows removed due to reproductive failure, 27 (45.0%) were removed during their first pregnancy, 29 (48.3%) during their second pregnancy, and 4 (6.7%) during their third pregnancy. The total rate of removal from the herd (*p* = 0.058) and the percentage of cows removed from the herd on account of reproductive failure (*p* = 0.245) did not differ significantly between pregnancies.

### 3.5. Neosporosis Impact on Reproduction Parameters and Performance

The rate of multiple inseminations did not differ in relation to *Neospora* serological status. The rate of removal from the herd was higher in seropositive or borderline cows than in seronegative cows (*p* < 0.001) but was not significantly different between high and low antibody titers (*p* = 0.487). The rate of removal from the herd due to reduced fertility, although higher in pregnancies with high antibody titers, did not differ significantly between serological groups (Table 7).

The outcome of pregnancy was available for 1238 of 1342 pregnancies, in which blood samples were collected from 1016 cows. Of these cows, 797 were sampled during one pregnancy, 216 during two pregnancies and 3 during three pregnancies. Out of 420 pregnancies in which cows had high anti-*Neospora* antibody titers, 193 were vaccinated during their pregnancy. The total abortion rate within unvaccinated pregnancies was 7.8% (82 of 1045 pregnancies, Table 8). Abortion rates in unvaccinated pregnancies significantly correlated with their antibody titer, were higher in cows with borderline antibody titers than in seronegative cows (*p* = 0.008), and were significantly higher in cows with high antibody titers than in ones with low antibody titers (*p* < 0.001, Table 7).

### 3.6. Variations Between Farms

The rate of abortions in different farms ranged between 6.6 and 19.8% of unvaccinated cows. The rate of abortion was associated with the prevalence of neosporosis at each farm (*p* < 0.001, Table 9). The rates of multiple inseminations, removal from the herd and removal due to reduced fertility did not differ significantly between farms (Table 8).

### 3.7. The Effect of Vaccination on Reproductive Performance

In an evaluation of the overall effect of vaccination in pregnancies in which antibody titers of 1:800 or higher were measured, no significant difference between vaccinated and unvaccinated cows was detected within abortion rates (*p* = 0.607), multiple inseminations (*p* = 0.744), vertical transmission (*p* = 0.445) or removal from the herd for any reason (*p* = 0.305), or for reasons relating to reproductive performance (*p* = 0.651) (Table 7).

A comparison of the abortion rates in vaccinated and vaccinated cows revealed lower abortion rates in the entire vaccinated group, and in two of the farms. However, none of these differences were statistically significant. The overall vaccine efficacy in the study groups was 10.4%, and the vaccine efficacy in different farms ranged between 54% and −167%. The efficacy of vaccination in preventing abortion was not significant in any of the farms (Table 9).

Of the 173 cows that were vaccinated as heifers, 23 were re-vaccinated during their second pregnancy. Although 94 of the 351 heifers with high antibody titers were re-sampled, in some cases, there was a decrease in the antibody titer in the second pregnancy, which made them ineligible for vaccination. There was no significant difference in the abortion rates between the cows that were sampled at least twice and were vaccinated once or twice (over two consecutive pregnancies) (*p* = 0.946, Table 10).

## 4. Discussion

The main aim of this study was to evaluate the long-term efficacy of anti-*Neospora* vaccination during consecutive pregnancies in naturally infected dams. The overall efficacy of either single vaccination or re-vaccination in preventing abortions was not significant. However, the data collected during this study provide detailed observations into the epidemiology of neosporosis in endemic farms.

The overall prevalence of neosporosis in the study population was 60.8% (95% CI: 57.8–63.77), which was higher than previous reports from Israel (44.3% [9], 41.4% [5]), perhaps since highly endemic farms were targeted for participation in this study. However, the prevalence of 33.1% of cows with antibody titers of 1:800 or higher (95% CI: 30.1–36.1%) was similar or slightly higher than previous reports from the area (35.5% [5], 25.1% [9]).

It has been shown in previous studies, as well as in the current one, that there is a positive correlation between anti-*Neospora* antibody titers and the risk of abortion [5,9,28]. Previously, a titer of 1:200 was not associated with an increased risk of abortions [5,9,28], and therefore, in several studies, as well as in the current and previous vaccine trials, a titer of 1:200 was considered as “borderline”, and the “clinically relevant” titer was set at 1:800.

The clinical significance of *Neospora* carriage is associated with an increased risk of abortions, but also the risk of vertical transmission. Vertical transmission is considered as the main route of transmission in endemic herds [7,29,30], and is therefore crucial for the maintenance of neosporosis within a population. Hence, the rate of potential vertical transmission should be taken into consideration in the implementation of control programs. Several previous studies have demonstrated that the rate of vertical transmission is higher in cows with high antibody titers than in cows with “borderline” or low titers [29,30], and may be up to 95%. Similar findings were recorded in this study, where a significant positive correlation was found between the dam and calf’s antibody titers (ρ = 0.679, *p* < 0.001) based on a relatively large sample size (N = 136). On one hand, these findings strengthen the conclusion that an antibody titer of 1:800 is, indeed, more clinically significant both regarding abortion rates and vertical transmission. However, on the other hand, 16 of 43 calves (37.2%) born to dams with antibody titers of 1:200 were born seropositive, most of which had high titers (10 of the 16). Therefore, these “borderline” cows, although not prone to abortion, may still contribute to the circulation of neosporosis in the herd. This contribution should be taken into consideration when constructing control programs in infected herds, and the selection of a testing method should be made accordingly.

In this study, we aimed to assess whether *Neospora* carriage may also affect fertility by estimating its association with multiple inseminations. Failure to conceive may be attributed to numerous factors which may either affect the chance to conceive, affect the chance to maintain pregnancy, or reflect early embryonic death. An evaluation of 1045 pregnancies did not reveal any significant association between *Neospora* serological status and the risk of multiple inseminations. These findings concur with the hypothesis that the dam’s cellular immunity is efficient during this stage of pregnancy and that infection or the re-emergence of parasites during this stage are unlikely to result in early abortion, as well as that most *Neospora*-related abortions occur in the second trimester, when cellular immunity is suppressed [11,31], in contrast to recent results from Brazil [32].

Similarly to previous studies [9,25], abortion rates increased in relation to the number of pregnancies, regardless of the *Neospora* serological status, with higher rates of abortions in older cows. Nevertheless, abortion rates were higher in seropositive cows, regardless of the number of pregnancies. Similarly, the chance of multiple insemination was higher in the second pregnancy than in the first, although the difference did not remain significant in later pregnancies. It appears that neosporosis in the herd affects both younger and older cows, and its influence does not subside with age [5].

Most cows maintained their serological status during consecutive pregnancies; however, fluctuations in antibody titers were observed. The incidence of horizontal transmission was evaluated with the inspection of the serological status of individual cows in consecutive pregnancies. Seroconversion was detected in 36.1% of seronegative cows; however, only a few (4 of 53) had high antibody titers. These cases with low antibody titers may be new infections in early stages or may reflect fluctuations in the antibody titers in cows with low antibody levels, which set them below the cutoff for detection, as also seen in the numerous cases of cows with a titer of 1:200 that converted to negative. In addition, some new infections in naïve cows may be resolved without resulting in their transformation to a carrier state. In total, only 13 of 144 seronegative or “borderline” cows had an antibody titer of 1:800 or higher in a future pregnancy, an incidence of 9% of horizontal transmission.

Almost 20% of the study population was removed from the herd during the study period. Cows were removed for a variety of reasons, with 29.4% due to reasons relating to reproductive performance. Although abortion rates increased with the number of pregnancies, neither the total rate of removal nor removal due to reproductive failure differed significantly between pregnancies. Interestingly, the rate of removal from the herd was higher in the seropositive than in the seronegative cows, regardless of their antibody titers. This rate may have been influenced by the serological testing during this study, since the farm owners were not blinded to the serological test results during the study. This selective removal (whether intentional or not) may have influenced the results of this study, reducing the sample size in consecutive pregnancies, especially in the group of positive, unvaccinated cows.

Vaccination is the main approach to control neosporosis, since no effective treatment is currently available, with increasing research and vaccine trials [33]. Several inactivated and attenuated vaccines have been developed against neosporosis [11,14,16,17,20,21,22,23,25,34,35]; however, the use of live parasites of less pathogenic or attenuated strains is considered as the more suitable approach in order to induce the cellular immunity required to effectively eliminate infection. A number of vaccine trials have shown promising effects in preventing abortions following infection during pregnancy [14,21,22,23,25]; however, the efficacy of vaccination in cases of the re-emergence of parasites in carrier animals is uncertain. Vaccine trials carried out under field conditions in endemic herds demonstrated relatively low and inconsistent efficacy, with considerable variation between farms [9,23].

In the current study, the efficacy of vaccination in preventing abortions was 10.4%, lower than in the previous trials using the same vaccination protocol (39% [25] and 28.4% [9]). Vaccination did not affect the antibody titers in the following pregnancies, and did not reduce the risk of vertical transmission. Vaccine efficacy varied considerably between farms, similarly to a previous trial [9]. In all farms, neosporosis was associated with abortion rates, while multiple insemination rates and removal from the herd did not differ significantly among farms. Vaccine efficacy in different farms varied between 54 and −167% and was not significant in any of the farms. Interestingly, the farm (Farm 5) that demonstrated a significant vaccine efficacy of 75% (*p* = 0.005) in a previous study [9] had slightly higher abortion rates in the vaccinated group in this study (VE = −5%). These results may suggest that farm management is not the only contributing factor to the variation in vaccine efficacy and that other environmental or health events may be involved, such as the presence of other infectious diseases or horizontal transmission events. It has been suggested that there might be a concurrent effect between *Neospora* and bovine viral diarrhea infections, which would lead to a higher chance of co-infection and of related abortions [36]. During the study period of the current study, there were reported cases of bovine ephemeral fever and an abortion wave due to the introduction of neosporosis into the heifer group in Farm 5. Although these events did not directly affect the study group, the circulation of other infectious diseases in the farm may have had some influence on the immunity of the herd, changing the equilibrium between the parasites and immune response of individual cows, leading to increased abortions and/or vertical transmission rates, which could not be prevented by vaccination.

The use of live vaccines leads to infection with attenuated strains, which should induce protective immunity against more virulent strains. The disadvantage of live attenuated vaccines is the risk of infecting naïve animals, which may also increase the rate of vertical transmission in the herd. Most vaccination trials did not address this issue; however, one study did report an increased occurrence of vertical transmission in vaccinated animals [23]. In the current study, no difference was found between the rate of vertical transmission and the calves’ antibody titers in vaccinated versus unvaccinated cows (*p* = 0.698). The fact that vaccination did not increase the risk of vertical transmission arises from the study design, in which only seropositive cows were vaccinated. Hence, there was no chance of seroconversion, which could lead to vertical transmission due to vaccination. However, since increased protection during the critical stage of pregnancy should, theoretically, also reduce the rate of vertical transmission (since both abortion and the delivery of an infected calf are the possible results of in utero infection of the fetus), this type of protection was not observed, but may coincide with the non-significant effect of vaccination on abortion in this study.

One of the aims of this study was to compare the efficacy of a single vaccination regime with repeated vaccination during subsequent pregnancies. Unfortunately, this aim was not achieved since the efficacy of the single vaccination was not significant. Therefore, the duration of protection could not be evaluated. Since this study was conducted under field conditions with no interference with farm management, there was some loss of follow-up and a substantial rate of removal from the herd. In addition, an outbreak of bovine ephemeral fever occurred in Israel during the study period and affected some of the farms. These limitations, which made the results more difficult to interpret, also represent the actual conditions affecting the use of vaccination in the field and highlight the complexity of the epidemiology of neosporosis in endemic areas. This complex epidemiology is the reason for the limited effectiveness of past vaccines when used in endemic herds [20,21,33].

## 5. Conclusions

The results of this study suggest that the vaccination of carrier cows mid-pregnancy is not a sufficient measure to control neosporosis in endemic herds. The balance between parasite re-emergence and immune response during pregnancy may be affected by numerous environmental and infectious factors, which cannot be overcome by vaccination alone. In addition, vertical transmission rates in vaccinated cows and in cows with low antibody titers (which may not be detected in all testing protocols) should be considered when constructing and implementing control programs in endemic herds.

## Figures and Tables

**Table 1:** *Neospora* seroprevalence in heifers sampled on days 110–120 of their first pregnancy in the five farms included in this study, and the number of vaccinated heifers at each farm.

Farm	Negative	Borderline (1:200)	Positive (≥1:800)	Vaccinated	Total
Farm 1	22 (17.1%)	37 (28.7%)	70 (54.3%)	33	129
Farm 2	70 (32.7%)	58 (27.1%)	86 (40.2%)	45	214
Farm 3	32 (21.3%)	24 (16.0%)	94 (62.7%)	46	150
Farm 5	231 (51.3%)	139 (30.9%)	80 (17.8%)	40	450
Farm 6	60 (51.7%)	35 (30.2%)	21 (18.1%)	9	116
Total	415 (39.2%)	293 (27.7%)	351 (33.1%)	173	1059

**Table 2:** *Neospora* serological titer in 198 unvaccinated cows sampled over two consecutive pregnancies, measured on days 110–120 of the first pregnancy of the heifers (rows), and again during their following pregnancy (columns).

		Second Pregnancy			
		0	1:200	≥1:800	Total
**First pregnancy**	**0**	53	26	4	83
	**1:200**	36	16	9	61
	**≥1:800**	8	9	37	54
	**Total**	97	51	50	198

**Table 3:** *Neospora* antibody titer in 11 unvaccinated cows sampled on days 110–120 over three consecutive pregnancies. The intensity of the background color reflects the serological status (negative, borderline or high antibody titer).

	Pregnancy		
Cow No.	1	2	3
2761	3200	800	800
2781	200	0	0
2798	200	3200	800
2802	800	200	0
2852	3200	200	800
2882	3200	800	3200
2900	0	0	0
7231	200	200	800
7255	800	0	3200
7270	3200	200	3200
7305	800	800	800

**Table 4:** Vertical transmission of neosporosis from dams to their calves. The association between the serological status between dams and their newborn calves prior to the ingestion of colostrum is described.

Dam Serological Status	Calf Serological Status							
	0	1:200	1:800	1:3200	1:12,800	Total	Positive ≥1:200 (%)	Positive ≥1:800 (%)
**0**	30	1	0	0	0	31	3.2	0
**1:200**	27	6	1	5	4	43	37.2	23.2
**1:800**	5	2	4	12	11	34 *	85.3	79.4
**1:3200**	3	1	2	12	7	25 **	88.0	84.0
**1:12,800**	0	0	0	2	1	3 ***	100	100
**Total**	65	10	7	31	23	136	52.2	44.8

As no significant differences were observed in the vertical transmission of vaccinated and unvaccinated dams, this Table includesall tested calves, of which * 15/34, ** 11/25 or *** 3/3 were of vaccinated dams.

**Table 5:** The number of inseminations required to achieve pregnancy in the first four pregnancies of 1059 cows in five farms during the study period. Comparisons between the rates of multiple inseminations (≥3) in each pregnancy are presented as odds ratios (ORs) with 95% confidence intervals (95% CIs), and their statistical significance (Fisher’s exact).

Pregnancy	1–2	≥3	Total	OR (95% CI)	*p*
**1**	658	380 (36.6%)	1038	ref	Ref
**2**	419	425 (50.4%)	844	1.76 (1.46–2.12)	<0.001
**3**	82	55 (40.1%)	137	1.16 (0.81–1.67)	0.421
**4**	3	2 (40.0%)	5	0.87 (0.08–9.58)	0.906
**Total**	1162	862 (42.6%)	2024		

**Table 6:** The outcome of 1475 pregnancies of 1059 cows in five farms during the study period. A comparison between abortion rates according to the number of pregnancies is presented as odds ratios (ORs) with 95% confidence intervals (95% CIs), and their statistical significance (Fisher’s exact).

Pregnancy	Calving	Abortion	Total	OR (95% CI)	*p*
**1**	924	82 (8.5%)	1006	ref	ref
**2**	364	79 (17.8%)	443	2.45 (1.75–3.41)	<0.001
**3**	14	12 (46.2%)	26	9.66 (4.32–21.57)	<0.001
**Total**	1302	173 (11.7%)	1475		

**Table 7:** The rate of abortions, multiple inseminations (three or more) and removal from the herd in 1238 bovine pregnancies according to their *Neospora* serological status and vaccine status during pregnancy. The statistical significance of the difference between serological groups and between vaccinated and unvaccinated cows with high serological titers (1:800 or higher) was evaluated using Fisher’s exact test and is presented for each parameter along with the OR and 95% CI, when relevant.

Antibody Titer	N	Abortions	OR (95% CI)	*p*	Multiple Inseminations	*p*	Removed from Herd	OR (95% CI)	*p*	Removed Due to Reduced Fertility	*p*
**0**	483	15 (3.1%)	Ref	ref	201 (41.6%)	ref	24 (5.0%)	ref	ref	6/24 (25.0%)	ref
**1:200**	335	25 (7.5%)	2.52 (1.31–4.85)	0.006	139 (41.5%)	0.972	33 (9.9%)	2.09 (1.21–3.61)	0.008	4/33 (12.1%)	0.215
**≥1:800** **Un-Vac**	227	42 (18.5%)	7.08 (3.84–13.08)	<0.001	89 (39.4%)	0.573	27 (11.9%)	2.58 (1.45–4.58)	0.001	14/27 (51.9%)	0.054
**≥1:800** **Vac**	193	32 (16.6%)	0.88 (0.53–1.45)	0.607 *	73 (37.8%)	0.744	17 (8.8%)	0.72 * (0.37–1.36)	0.305 *	10/17 (58.8%)	0.651 *
**Total**	1238	114 (9.2%)			502 (40.6%)		101 (8.2%)			34/101 (33.7%)	

* Compared with ≥1:800 unvaccinated dams.

**Table 8:** The rates of cows seropositive for neosporosis at an IFAT titer of 1:200, abortions, multiple inseminations (three or more) and removal from the herd in 1045 bovine pregnancies in the five dairy farms that participated in this study. The significance of the difference between farms using the univariable GEE test, with the cow defined as within subject effect, appears at the bottom of each column. Multiple comparisons between the farms are presented for parameters with significant associations, along with their significance through their OR and 95% CI.

Farm	N	Seropositive or Borderline Titer (1:200)	OR (95% CI)	*p*	Abortions	OR (95% CI)	*p*	Multiple Inseminations	Removed from Herd	Removed Due to Reduced Fertility
**Farm 1**	126	97 (77.0%)	4.20 (2.39–7.39)	<0.001	25 (19.8%)	3.5 (1.45–8.46)	0.005	62 (49.6%)	14 (11.1%)	2/14 (14.3%)
**Farm 2**	244	140 (57.4%)	1.69 (1.07–2.68)	0.025	16 (6.6%)	0.99 (0.39–2.45)	0.987	89 (36.5%)	16 (6.6%)	7/16 (43.8%)
**Farm 3**	119	85 (71.4%)	3.14 (1.81–5.45)	<0.001	15 (12.6%)	2.1 (0.79–5.21)	0.137	50 (42.0%)	9 (7.6%)	5/9 (55.6%)
**Farm 5**	450	193 (42.9%)	0.94 (0.62–1.44)	0.786	19 (4.2%)	0.62 (0.25–1.52)	0.300	186 (41.3%)	39 (8.7%)	9/39 (23/1%)
**Farm 6**	106	47 (44.3%)	Ref		7 (6.6%)	ref		42 (39.6%)	6 (5.7%)	1/6 (16.7%)
**Total**	1045	562 (53.8%)			82 (7.8%)			429 (41.1%)	84 (8.0%)	24/84 (28.6%)
**Sig**		<0.001			<0.001			0.2	0.499	0.145

**Table 9:** The rate of abortions in the five dairy farms that participated in this study in cows that were seropositive for neosporosis with a titer of at least 1:800 according to their vaccination status against neosporosis during pregnancy using live frozen vaccination. The statistical significance (chi-square test) of the association between vaccination status and abortion rate, along with the vaccine efficacy (VE), are specified for each farm.

Farm	N Unvaccinated	Abortions (%)	N Vaccinated	Abortions (%)	*p*	VE
**Farm 1**	49	14 (28.6%)	40	8 (20.0%)	0.351	30%
**Farm 2**	65	10 (15.4%)	56	4 (7.1%)	0.158	54%
**Farm 3**	57	11 (19.3%)	46	12 (26.1%)	0.411	−35%
**Farm 5**	44	6 (13.6%)	42	6 (14.3%)	0.931	−5%
**Farm 6**	12	1 (8.3%)	9	2 (22.2%)	0.479	−167%
**Total**	227	42 (18.5%)	193	32 (16.6%)	0.700	10.40%

**Table 10:** The rate of abortions in cows that were sampled during at least two pregnancies and that were seropositive for neosporosis with a titer of at least 1:800 as heifers, according to the number of pregnancies during which they were vaccinated against neosporosis using live frozen vaccination.

	N	Abortions (%)	*p*
**Unvaccinated**	49	8 (16.3%)	ref
**Single vaccination**	35	7 (20.0%)	0.775
**Double vaccination**	23	4 (17.4%)	1
**Total**	107	19 (17.8%)	

## Data Availability

The data presented in this study are available on request from the corresponding author.
